# Intergenerational Effects of Paternal Diets on Offspring Health and Reproduction

**DOI:** 10.3390/nu18183008

**Published:** 2026-09-14

**Authors:** Lirong Geng, Jiabin Zhang, Guitian He

**Affiliations:** College of Animal Science and Technology, Tarim University, Aral 843300, China

**Keywords:** paternal diet, reproductive ability, embryonic development, cognitive ability, offspring

## Abstract

Fat, protein, and micronutrients serve as essential components of human nutrition, and their balanced intake is critical for maintaining health. Chronic nutritional imbalances may adversely affect not only individual health but that of subsequent generations. Accumulating evidence indicates that paternal nutritional status before conception contributes to offspring health through diet-induced alterations in sperm quality and epigenetic programming. The genetic integrity and physiological condition of sperm are susceptible to long-term dietary exposures, providing a potential biological basis for paternal nutritional programming. This review summarizes current evidence regarding the effects and potential molecular mechanisms by which paternal dietary patterns, including high-fat diet (HFD), low-protein diet (LPD), and folic acid (FA) intake, influence reproductive competence, embryonic development, and cognitive function in offspring. Notably, most available evidence is derived from animal models, whereas corresponding human data remain limited. Current findings suggest that paternal dietary patterns may influence reproductive, embryonic, and offspring outcomes through interconnected biological pathways involving sperm quality and epigenetic regulation, although the underlying mechanisms have not been fully elucidated. We also discuss potential intervention strategies evaluated in preclinical studies and highlight current knowledge gaps and future research priorities for understanding the intergenerational effects of paternal nutrition.

## 1. Introduction

**Figure 1:**
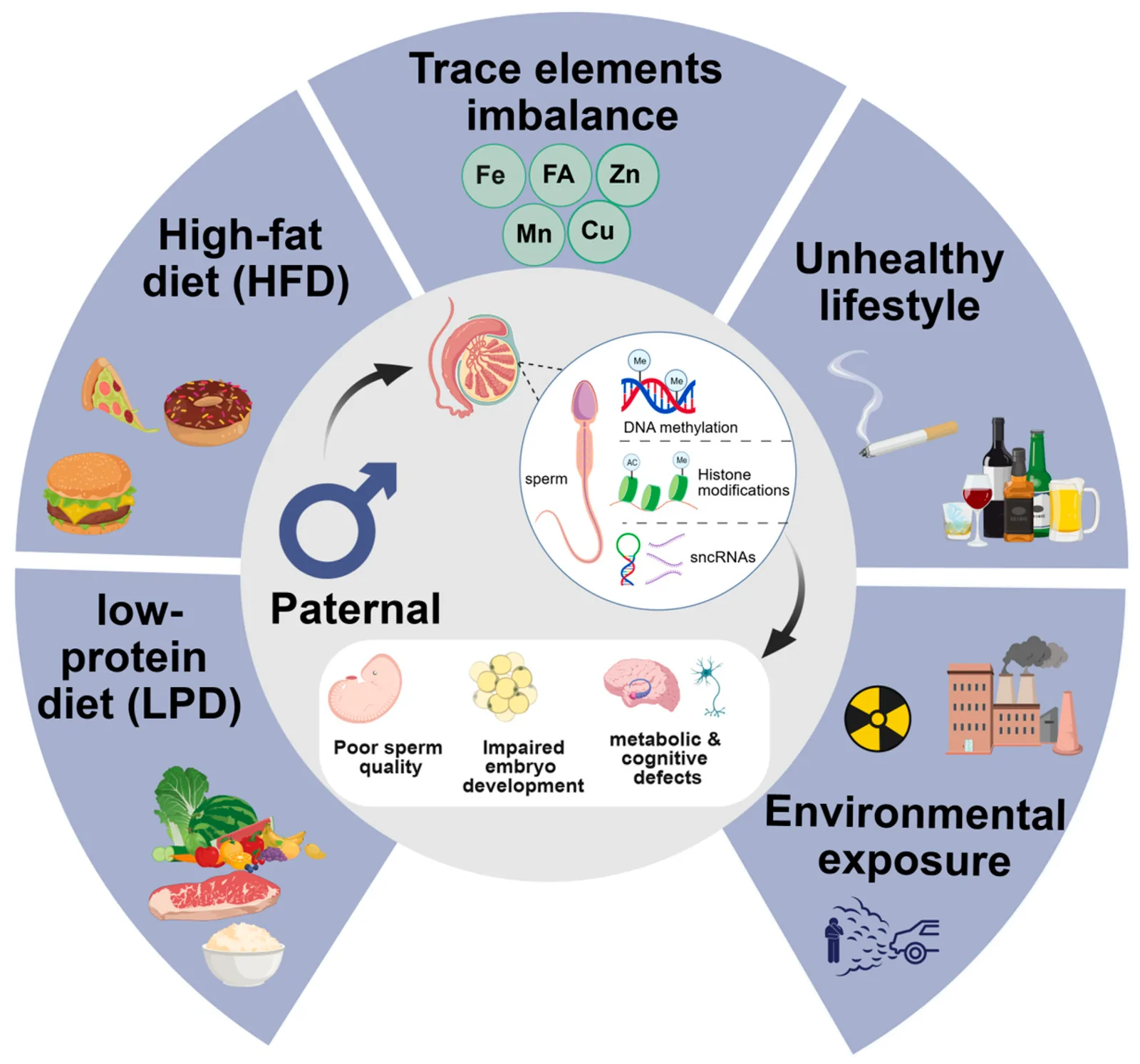
Overview of the potential intergenerational effects of paternal nutritional and environmental exposures on offspring health and reproductive development. Paternal nutritional and environmental exposures may alter paternal metabolic and reproductive status, leading to changes in sperm quality and epigenetic information. These paternal molecular alterations may subsequently influence embryonic and placental programming, potentially affecting offspring growth, metabolism, reproductive development, and neurodevelopmental or behavioral outcomes. Arrows indicate the direction of established mechanisms or biological processes.

Nutritional factors, in conjunction with genetic inheritance, significantly influence offspring through parental effects [1,2]. The influence of maternal diet on offspring development has been extensively documented. Specifically, macronutrients, such as proteins and fats, have an important impact on offspring health. Consumption of diets low in protein or high in fat during pregnancy has been associated with metabolic abnormalities and neurodevelopmental alterations in offspring [3]. In addition, imbalances in trace element intake, including deficiency or excess, have been associated with fetal neurological defects and intrauterine growth restriction, as well as impacting birth weight, blood glucose concentration, and insulin resistance in offspring [4]. However, increasing evidence from epidemiological and experimental studies indicates that paternal diets in the preconception period also play an important role in the metabolic health of offspring [5]. An unhealthy paternal lifestyle, including a high-fat diet (HFD), low-protein diet (LPD), alcohol consumption, and smoking, has been associated with reduced fertility or infertility [6,7,8,9]. In addition, long-term exposure to environmental hazards, such as air pollution or radiation has also been associated with reproductive disorders [10,11,12], highlighting paternal gametic quality as an important contributor to embryonic viability and long-term offspring health [4] (Figure 1). To systematically examine the molecular and developmental consequences of paternal diet, this review focuses on three representative nutritional patterns HFD, LPD, folic acid (FA) intake, and synthesizes current evidence regarding their effects on male reproductive competence, embryogenesis, and cognitive function in the offspring (Figure 1).

Over the past few years, several comprehensive reviews have summarized the role of paternal nutrition and sperm epigenetic remodeling in intergenerational inheritance. These reviews have primarily focused on the epigenetic mechanisms by which paternal environmental exposures influence offspring phenotypes, including DNA methylation, histone modifications, chromatin remodeling, and sperm-borne small non-coding RNAs (sncRNAs), or have broadly discussed paternal lifestyle factors such as diet, obesity, stress, exercise, and environmental toxicants [2,13,14]. Although these reviews have substantially advanced our understanding of paternal epigenetic inheritance, they generally emphasize individual epigenetic pathways or specific paternal exposures rather than systematically comparing different nutritional patterns across successive developmental stages. This review addresses this gap by integrating three representative paternal dietary patterns, HFD, LPD, and FA intake, across reproductive function, embryonic development, and offspring health, with emphasis on shared and diet-specific mechanisms, evidence strength, translational challenges, and potential interventions.

Paternal dietary patterns and their impacts on offspring health, with particular attention to high-fat diet, low-protein diet, and folic acid intake, are a broad and evolving area of research and the focus of this narrative review. Relevant literature was identified through systematic searches of PubMed, for studies published up to January 2026 using combinations of the following keywords: “paternal diet”, “paternal nutrition”, “high-fat diet”, “low-protein diet”, “folic acid”, “folate”, “sperm epigenetics”, “DNA methylation”, “histone modification”, “small non-coding RNA”, “offspring health”, “embryonic development”, and “intergenerational inheritance”. Original research articles and relevant review articles published in English were considered. Priority was given to studies investigating paternal preconception dietary exposures and their effects on sperm biology, embryonic development, reproductive outcomes, and offspring health.

## 2. HFD

### 2.1. Effects of Paternal HFD on Paternal and Offspring Reproductive Function

Obesity and overweight are major global health challenges and have been associated with impaired male reproductive health [15]. In men, obesity has been consistently associated with reduced fertility potential, and epidemiological studies have reported that overweight and obesity are associated with impaired semen quality, including reduced sperm concentration, motility, normal morphology, acrosome reaction, increased sperm DNA damage, and lower pregnancy or reduced fertility potential [16,17,18,19]. However, these findings are primarily derived from observational studies and therefore demonstrate associations rather than direct causal relationships. Moreover, obesity is a complex metabolic condition influenced by multiple genetic, environmental, and lifestyle factors, and should not be considered equivalent to dietary exposure alone.

**Figure 2:**
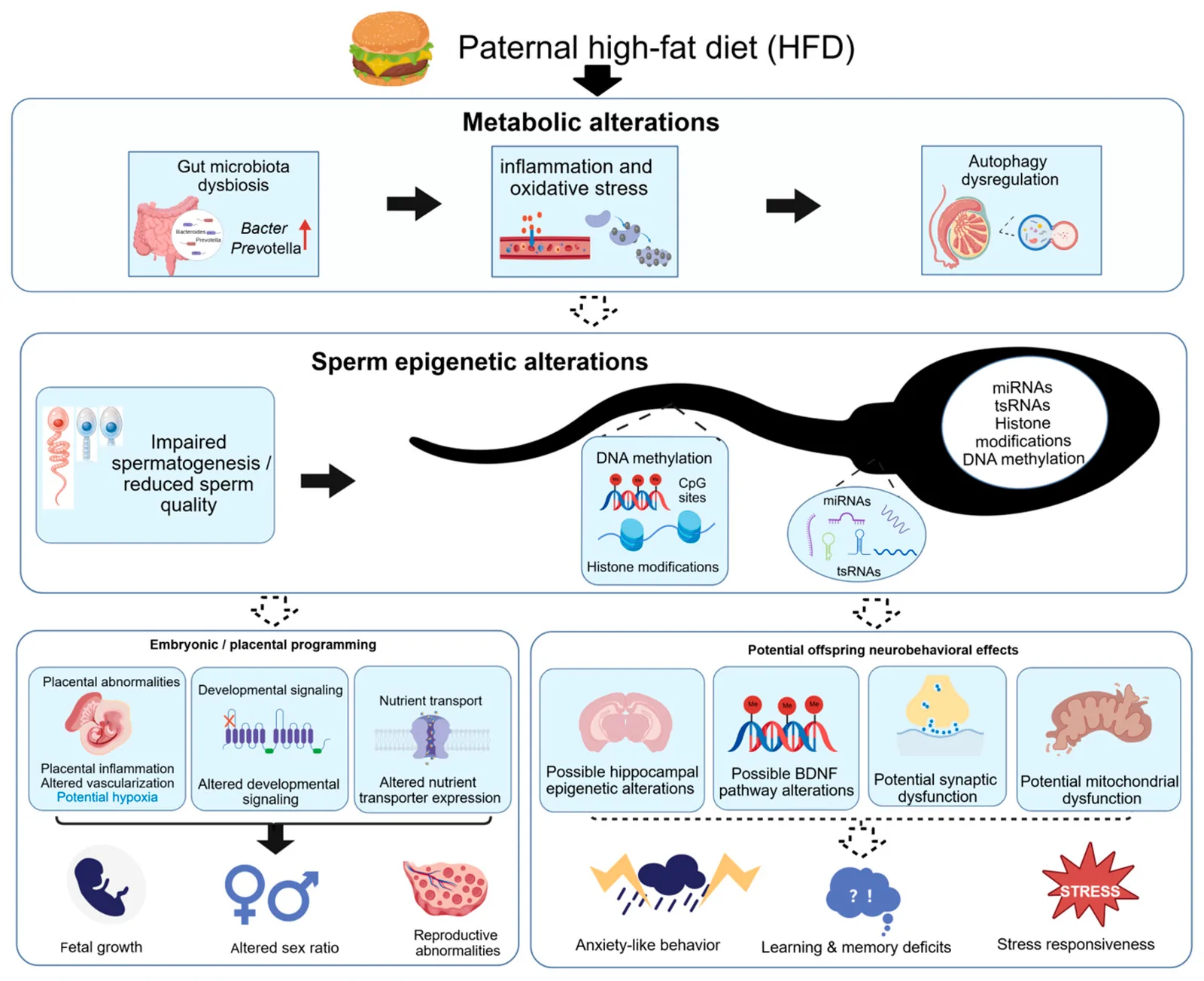
Proposed pathways linking paternal high-fat diet (HFD) exposure to intergenerational effects on offspring health and reproduction. Paternal HFD may induce metabolic and oxidative disturbances that are associated with impaired spermatogenesis and altered sperm epigenetic information. These alterations may subsequently influence embryonic/placental programming and potentially contribute to offspring reproductive and neurobehavioral phenotypes. Solid arrows indicate established mechanisms or the direction of the biological process, whereas dotted arrows indicate proposed mechanisms that remain unconfirmed. Red arrows indicate promotion or upregulation, while blue arrows indicate inhibition or downregulation.

Paternal HFD exposure and obesity are related but distinct. HFD represents an experimental dietary intervention, whereas obesity is a metabolic phenotype that may develop following prolonged HFD exposure and is influenced by species, genetic background, diet composition, and exposure duration. Therefore, obesity-related human evidence is presented as clinical context rather than direct evidence of HFD effects. Most evidence on the intergenerational effects of paternal HFD comes from experimental animal models, predominantly mice, whereas human evidence remains limited and largely observational. Thus, animal findings should not be directly extrapolated to humans. Throughout this section, epidemiological evidence on paternal obesity is distinguished from experimental evidence on paternal preconception HFD exposure. In animal models, paternal HFD exposure has been associated with metabolic disturbances, including weight gain, metabolic dysfunction, insulin resistance, and impaired glucose homeostasis [20,21,22]. Furthermore, HFD has been associated with impaired spermatogenesis, alterations in gut microbiota, and sperm epigenetic changes, including altered *SETD2* gene methylation in F0 and F1 mice [23,24]. Mechanistically, HFD was accompanied by a significant increase in the genera *Bacteroides* and *Prevotella* within the gut microbiome of mice, leading to elevated endotoxin levels, dysregulation of testicular gene expression, and localized epididymal inflammation (Figure 2). These conditions collectively contribute to defects in spermatogenesis and reduced sperm motility [23]. Recent evidence indicates that HFD exacerbates lead exposure-induced impairments in spermatogenesis in mice, with the gut microbiota playing a critical role in this process [25]. Notably, probiotic supplementation has shown potential for mitigating these adverse effects [25]. Current studies in mice suggest that probiotic supplementation may represent a potential experimental strategy for restoring the gut microbiota ecosystem and enhancing spermatogenic function [25,26,27]. Furthermore, autophagy has been identified as a factor in the regulation of sperm survival and motility within the testes [28]. It has also been implicated in the impairment of spermatogenesis in mice with diet-induced obesity. The administration of chloroquine, an autophagy inhibitor, has been demonstrated to suppress autophagic activity, thereby improving spermatogenesis [29]. Several preclinical studies have explored dietary modification, probiotic administration [25,26,27], or exercise as potential approaches to counteract HFD-associated metabolic and reproductive alterations [30,31] (Table S2). However, these interventions remain experimental and require further validation before clinical application.

Controlled animal studies have demonstrated that paternal HFD exposure before conception, frequently accompanied by obesity or obesity-like metabolic alterations, is associated with impaired reproductive function and adverse offspring outcomes through epigenetic and other non-genetic mechanisms [32,33,34]. Under these experimental conditions, male mice fed HFD for several weeks before mating exhibited alterations in sperm quality, sperm epigenetic profiles, and offspring metabolic and reproductive phenotypes. However, because HFD-induced obesity was not consistently quantified across studies, the independent contributions of dietary fat intake and obesity remain difficult to distinguish. [32,33,34]. Multigenerational exposure to paternal obesity and obesogenic environments has been associated with reproductive alterations in subsequent generations [33]. In female offspring, paternal HFD disrupted normal estrous cycle patterns and resulted in a reduction in the number of primordial follicles as well as a decline in follicular reserves [34,35]. Additionally, paternal HFD elevated the expression of glucose transporters GLUT1, GLUT3, and GLUT4 in the ovaries of female offspring, with GLUT4 predominantly localized in cumulus cells. This was accompanied by increased accumulation of glucose and lipid metabolites in the cumulus–oocyte complex and delayed embryonic development [34] (Figure 2). In male offspring, paternal HFD significantly reduced sperm viability, body weight, and circulating testosterone and altered genes involved in steroid biosynthesis [35]. Notably, research has demonstrated that disruptions in the paternal gut microbiota can adversely affect offspring health, contributing to an increased risk of low birth weight, severe growth restriction, and premature mortality [36]. Conversely, the majority of studies have established that maintaining the homeostasis of gut microbiota is essential for reproduction [37]. These findings further suggest that HFD-induced alterations in the paternal gut microbiota may contribute to impaired reproductive function and offspring phenotypes, although the causal relationship remains to be fully established.

Several preclinical studies have investigated whether dietary modification or exercise before conception can attenuate the adverse reproductive effects associated with paternal HFD exposure. Evidence from paternal overnutrition models suggests that preconception interventions, including exercise and dietary modification, may partially ameliorate sperm abnormalities, epigenetic alterations, and offspring phenotypes [30,31,38] (Table S2). However, these findings remain predominantly preclinical and require further validation. Although paternal exercise may also ameliorate HFD-induced sperm and offspring metabolic alterations, the evidence remains predominantly preclinical, and further clinical studies are needed to establish its relevance to prospective fathers [39]. One study investigated the combined effects of HFD and cadmium exposure on male reproductive function. However, because both factors exert complex and potentially interacting biological effects, the findings should not be interpreted as indicating a beneficial effect of cadmium under HFD conditions. The biological significance of these combined exposures remains uncertain and requires further investigation [40].

### 2.2. Effects of a Paternal HFD on Embryonic Development

Paternal HFD exposure has been associated with placental inflammatory changes and altered placental nutrient transporter expression in animal models [31]. In mice, paternal HFD exposure for 3 months significantly reduced placental and fetal tissue weights in male offspring and altered placental nutrient transporter mRNA expression in female placentae, indicating sex-dependent effects on placental function [31]. Consistent with these findings, transcriptomic analysis of embryos fathered by obese males identified differential expression of genes associated with early embryonic development and metabolism, suggesting altered embryonic programming following paternal obesity [41]. Bernhardt et al. [41] demonstrated that paternal diet-induced obesity significantly altered the transcriptomic landscape of pre-implantation embryos, affecting genes involved in early embryonic development, metabolism, and cell differentiation, suggesting that paternal obesity may influence developmental programming from the earliest stages of embryogenesis. Among the differentially expressed genes, GATA6 and SAMD4B were upregulated predominantly in female embryos, suggesting potential sex-specific responses to paternal obesity [41]. Altered expression of these genes may be associated with later metabolic susceptibility in offspring; however, their functional roles in paternal obesity-induced developmental programming remain to be established [41]. Alterations in sperm DNA methylation-related pathways have been reported following paternal HFD exposure and may be associated with changes in developmental signaling; however, the contribution of specific pathways, including Wnt, Hedgehog, TGF-β, and Notch signaling, remains incompletely defined [41].

In one study, paternal consumption of an HFD before conception was associated with improved embryonic development following paternal exercise [31]. Part of the reason is that exercise can change the expression of miRNA in the sperm, downregulation of miR-193b inhibits the expression of tumor necrosis factor (TNF-α) in placental cells, then, TNF-α can be targeted to reduce the expression of SLC38A2, which plays an important role in placental and fetal development, and was associated with restoration of placental nutrient transport and offspring birth weight [31]. Nevertheless, effective strategies to mitigate the deleterious impact of paternal HFD on embryonic development remain to be elucidated.

### 2.3. Effects of Paternal HFD on Cognitive Function in Offspring

Animal studies have reported associations between paternal HFD exposure and offspring neurobehavioral alterations, potentially involving changes in hippocampal gene expression and epigenetic regulation. However, whether these alterations represent direct causal mechanisms requires further investigation.

A mouse study reported that paternal HFD exposure for 10 weeks before mating was associated with impaired cognitive performance in offspring and increased methylation of the hippocampal Brain-derived neurotrophic factor (BDNF) promoter in sperm and offspring hippocampus, together with alterations in BDNF/TrkB signaling [42]. These findings suggest a potential association between paternal sperm epigenetic alterations and offspring hippocampal function, although the causal contribution of BDNF methylation remains to be established.

High-fat feeding has been associated with altered hippocampal SNAP-25 expression and impaired synaptic plasticity in rodent models [43] (Figure 2). Whether similar changes are specifically induced by paternal HFD exposure and contribute to offspring neurobehavioral phenotypes remains to be established. Interestingly, axonal synthesis of SNAP-25 is required during neural development to properly form synapses and release synaptic vesicles from presynaptic terminals [43]. This suggests that a HFD may alter synaptic synthesis of SNAP-25, leading to impaired synaptic plasticity of offspring [43]. In rodent models, HFD exposure appears to negatively affect synaptic plasticity and electrophysiological responses in the hippocampus, a brain region crucially involved in information and emotion processing [43].

Collectively, current evidence supports an association between paternal HFD exposure, obesity-related metabolic dysfunction, and impaired reproductive and offspring health. Nevertheless, substantial heterogeneity exists among studies with respect to animal species, dietary fat composition, exposure duration, obesity severity, and experimental design. Consequently, it remains difficult to distinguish the independent effects of HFD from those of obesity, and the underlying causal mechanisms require further investigation, particularly in humans.

## 3. LPD

### 3.1. Effects of Paternal LPD on Paternal and Offspring Reproductive Function

Compared with paternal HFD, evidence regarding the effects of paternal LPD on offspring health remains relatively limited and is derived predominantly from experimental animal models. Therefore, findings from paternal LPD studies should be interpreted cautiously, particularly when considering their relevance to human populations. Moderate paternal malnutrition, particularly LPD exposure, has been associated with altered metabolic programming and impaired organ development in offspring [44]. Increasing evidence suggests that paternal protein restriction also affects male reproductive function through epigenetic and metabolic disturbances in the testis. Sperm from males exposed to an LPD exhibit global DNA hypomethylation accompanied by reduced testicular expression of DNA methyltransferases and key regulators of one-carbon metabolism. Specifically, decreased expression of DNA methyltransferases 1 (DNMT1) and DNA methyltransferases 3-like (DNMT3L), together with reduced levels of folate-cycle enzymes, including dihydrofolate reductase, methylenetetrahydrofolate reductase, and methionine reductase, has been reported [45]. Notably, supplementation with methyl-donors partially reversed these alterations by restoring seminiferous tubule morphology and reducing the expression of apoptosis-related genes such as *Bax*, *Csap1*, and *Fas* in the testis [45] (Table S2). A mouse study reported that paternal LPD was associated with altered expression of miR-451a, miR-200c, and miR-92a in the mammary tissues of female offspring, which was associated with altered AMPK-related metabolic pathways. However, these findings do not establish altered sperm miRNAs as the direct mediator of the offspring phenotype. Recent mouse studies showed that paternal LPD altered the sperm RNA landscape and preimplantation embryo developmental kinetics, raising the possibility that sperm-borne RNAs may contribute to paternal nutritional programming [44]. Direct evidence linking paternal LPD exposure to impaired reproductive function in offspring remains limited. Nevertheless, the metabolic disturbances associated with paternal protein restriction may indirectly compromise reproductive development and function in the next generation [46]. Collectively, these findings provide mechanistic insight into how paternal malnutrition shapes offspring health and reproductive capacity through epigenetic reprogramming.

### 3.2. Effects of Paternal LPD on Embryonic Development

**Figure 3:**
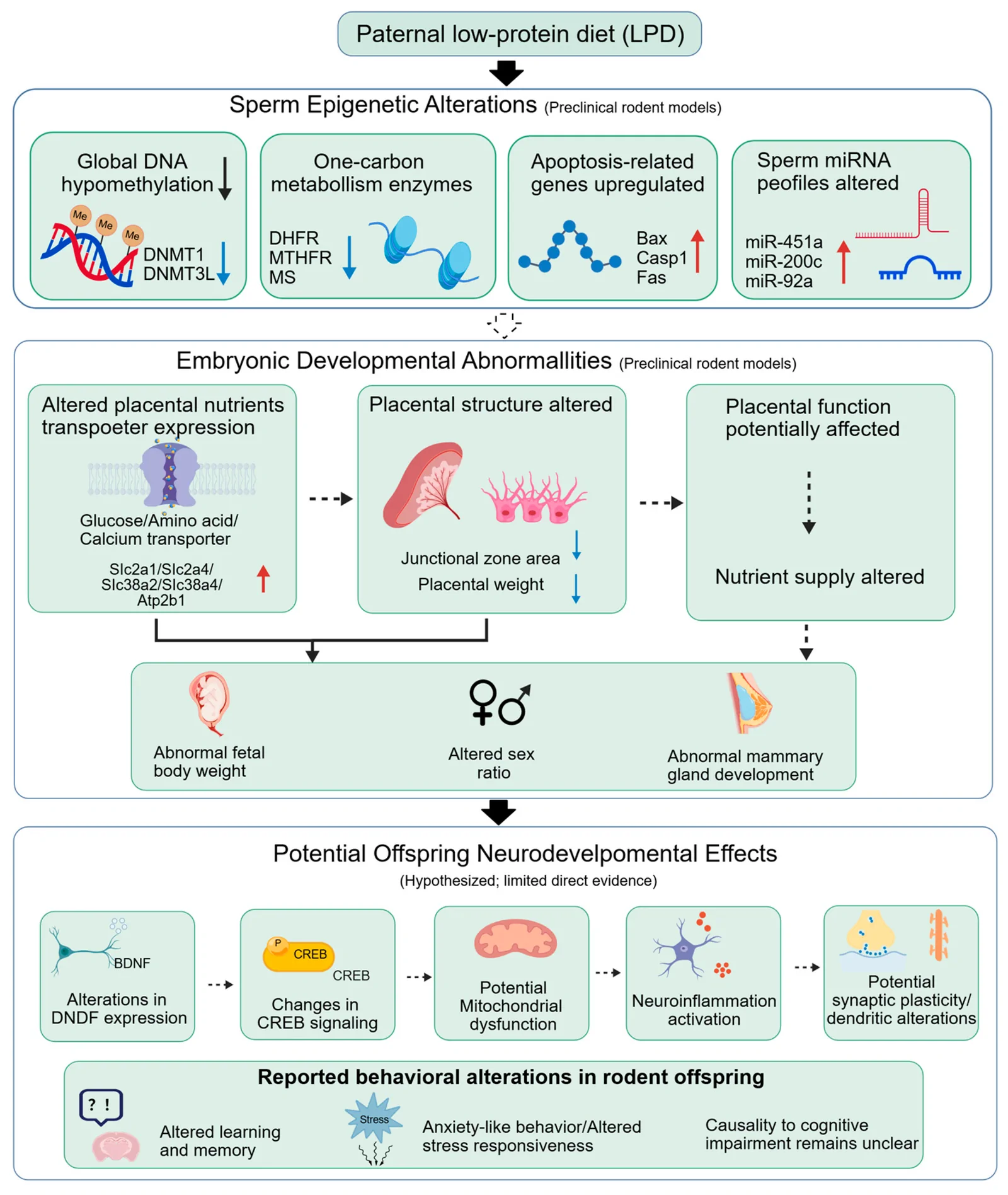
Proposed pathways linking paternal low-protein diet (LPD) exposure to intergenerational effects on offspring health and development. Paternal LPD may disrupt one-carbon metabolism and sperm epigenetic information, potentially affecting embryonic/placental nutrient programming and fetal development, with possible subsequent effects on offspring reproductive and neurobehavioral outcomes. Solid arrows indicate established mechanisms or the direction of the biological process, whereas dotted arrows indicate proposed mechanisms that remain unconfirmed. Red arrows indicate promotion or upregulation, while blue arrows indicate inhibition or downregulation.

Paternal LPD exposure has been linked to impaired fetal growth and adverse developmental outcomes in offspring, largely through alterations in sperm epigenetic status. Evidence from animal studies indicates that paternal protein restriction disrupts placental development and fetal nutrient supply (Table S1). In mice, paternal LPD increased fetal weight during late gestation while reducing placental weight [47]. In parallel, paternal LPD significantly reduced the male-to-female offspring ratio (*p* = 0.03) and increased male offspring birth weight (*p* = 0.009), with higher body weight persisting during the early postnatal period (*p* < 0.02) [48]. Paternal LPD altered placental nutrient transport by increasing the expression of glucose transporters (*Slc2a1* and *Slc2a4*), amino acid transporters (*Slc38a2* and *Slc38a4*), and the calcium transporter *Atp2b1*, changes that were accompanied by reduced placental junctional area and lower free fatty acid content [47] (Figure 3). Notably, methyl-donor supplementation partially ameliorated paternal LPD induced alterations in testicular physiology and normalized the expression of DNA methyltransferases in mice [45]. These findings suggest that methyl-donor availability may influence sperm epigenetic programming under paternal protein restriction [45]. Whether these changes directly improve placental development or offspring growth remains to be established. These findings further suggest that paternal protein restriction may influence offspring development through alterations in sperm epigenetic regulation; however, the available evidence is derived primarily from rodent models and requires further validation.

### 3.3. Effects of Paternal LPD on Offspring Cognitive Function

Evidence regarding the effects of paternal LPD on offspring cognitive function remains limited. Although some studies have reported behavioral or neurodevelopmental alterations following paternal nutritional imbalance exposure, direct evidence linking paternal preconception LPD to cognitive impairment remains insufficient. Indirect evidence from maternal and prenatal protein-restriction models suggests that inadequate protein availability may affect offspring neurodevelopment and cognitive function [49,50,51,52]. Current evidence suggests a potential association between paternal nutritional status and offspring neurobehavior across different species, but the underlying mechanisms and causal relationships remain unclear. These indirect studies have linked protein restriction to altered hippocampal development, reduced neurotrophic signaling, neurotransmitter dysregulation, and cognitive or behavioral alterations [49,50,51]. Nutritional interventions such as prenatal choline supplementation have partially ameliorated cognitive deficits in offspring exposed to prenatal protein restriction [52]. However, these findings provide indirect mechanistic context and cannot be directly extrapolated to paternal preconception LPD.

Direct evidence from paternal nutritional models remains limited. Recent mouse studies have shown that paternal undernutrition can alter the sperm RNA landscape and preimplantation embryo developmental kinetics, raising the possibility that sperm-borne molecular signals may contribute to paternal nutritional programming. However, the causal contribution of specific sperm RNAs to offspring neurodevelopment has not been established. Similarly, paternal dietary macronutrient imbalance has been associated with altered behavioral phenotypes in offspring, including anxiety-like behavior and changes in behavioral adaptability [53]. These findings indicate potential effects of paternal nutritional status on offspring neurobehavior but do not establish paternal LPD as a direct cause of cognitive impairment. Beyond behavioral outcomes, paternal nutritional imbalance has also been associated with alterations in offspring energy metabolism and mitochondrial function [9] (Figure 3). However, whether these metabolic changes contribute to offspring neurodevelopment following paternal LPD remains unclear.

Potential mechanisms may involve coordinated alterations in sperm epigenetic information, early embryonic programming, and offspring metabolic regulation. Changes in sperm RNA or other epigenetic signals could potentially influence early embryonic gene regulation and subsequent developmental trajectories. However, direct evidence linking these molecular alterations to offspring brain development or cognitive phenotypes following paternal LPD is currently lacking. Overall, current evidence is insufficient to conclude that paternal LPD directly was associated with impaired offspring cognitive function. Available studies suggest that paternal nutritional imbalance may influence offspring neurobehavior through sperm-mediated and developmental programming mechanisms, but these effects remain incompletely characterized. Differences in dietary composition, exposure duration, animal model, behavioral assessment, and experimental design further limit comparisons across studies. Future studies should directly examine paternal LPD, validate specific sperm-borne mediators, and determine whether the observed behavioral phenotypes are reproducible and causally linked to paternal nutritional exposure.

## 4. Folic Acid (FA)

### 4.1. Effects of FA in Paternal Diet on the Reproductive Ability of Self and Offspring

**Figure 4:**
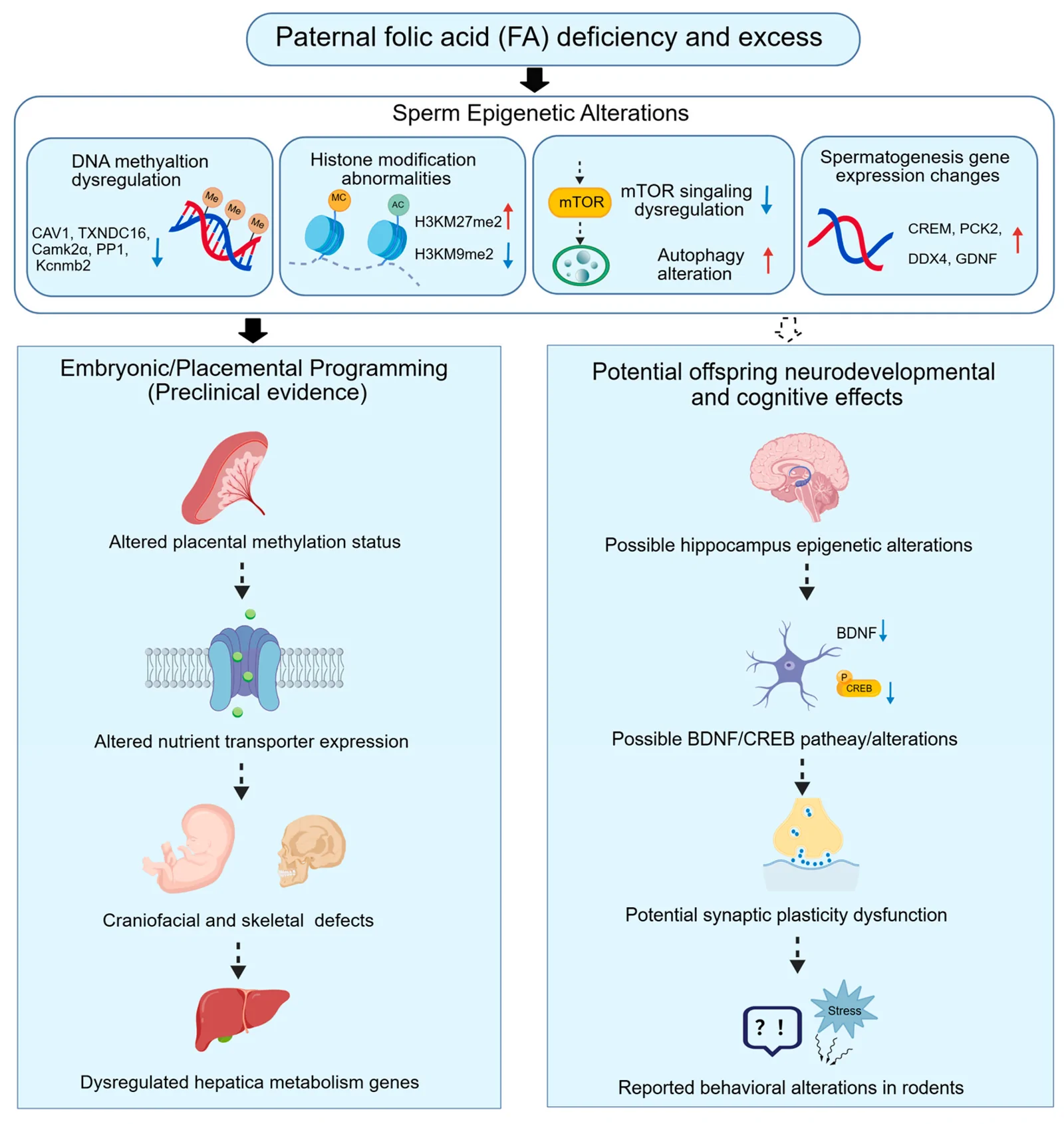
Proposed pathways linking paternal folic acid (FA) deficiency or excess to intergenerational effects on offspring health and development. Paternal FA deficiency or excess may disrupt one-carbon metabolism and sperm epigenetic regulation, potentially altering embryonic/placental programming and subsequent offspring reproductive, developmental, and neurobehavioral outcomes. Solid arrows indicate established mechanisms or the direction of the biological process, whereas dotted arrows indicate proposed mechanisms that remain unconfirmed. Red arrows indicate promotion or upregulation, while blue arrows indicate inhibition or downregulation.

Folate refers to naturally occurring forms of vitamin B9 found in foods, whereas FA is the synthetic oxidized form commonly used in supplements and fortified foods. In addition, methyl-donor supplementation (e.g., folate, choline, and methionine) represents a broader nutritional intervention that may influence one-carbon metabolism. Accordingly, the effects of these nutritional factors should be interpreted in the context of their chemical forms, doses, and exposure durations, with particular attention to the distinct effects of paternal FA deficiency and supplementation. As an important methyl-donor nutrient involved in one-carbon metabolism, FA contributes to the regulation of DNA, RNA, and protein methylation [54]. Paternal FA deficiency has been associated with altered sperm DNA methylation and dysregulated expression of placental genes, including *CAV1* and *TXNDC16* [54] (Figure 4).

These findings suggest that altered paternal FA status may affect reproductive outcomes through changes in sperm epigenetic programming (Table S1). Further evidence from male BALB/c mice showed that lifetime exposure to a FA-deficient diet containing 0.3 mg/kg FA significantly reduced sperm counts compared with the control diet (*p* < 0.05), accompanied by altered sperm epigenetic profiles, including increased inter-individual variation in methylation at the imprinted *H19* locus [55]. In contrast, paternal FA supplementation has produced variable effects depending on dose and experimental model. In the same mouse study, lifetime exposure to 20 mg/kg FA did not significantly reduce sperm counts, whereas supplementation at 40 mg/kg significantly reduced sperm counts compared with controls (*p* < 0.05) [55]. These findings suggest that the reproductive effects of paternal FA supplementation may depend on the magnitude of exposure rather than following a simple dose–response relationship (Table S2). In roosters, FA supplementation was associated with altered expression of spermatogenesis-related genes (*CREM*, *PCK2*, *DDX4*, and *GDNF*) and changes in mTOR and autophagy-related markers, including reduced p-mTOR and H3K27me2 and increased H3K9me2 expression, together with improved semen quality and spermatogenesis [56] (Figure 4).

Altered FA intake has been associated with changes in mTOR signaling and autophagy-related pathways in experimental models [56]. However, the mechanistic relationship among FA availability, mTOR signaling, autophagy, and reproductive outcomes remains incompletely understood. Therefore, these pathways should be considered potential mechanisms rather than established causal pathways, and further functional studies are required to determine their relative contributions to FA-associated reproductive effects.

Although similar epigenetic and metabolic pathways have been reported across animal models, the effects of paternal FA deficiency and supplementation are not fully consistent. Differences in species, dietary FA concentration, exposure duration, and experimental design may contribute to the variation in reported outcomes [55,56] (Table S1). Therefore, findings from rodent and avian models should be interpreted as complementary preclinical evidence rather than as directly equivalent biological effects or evidence for optimal paternal FA intake in humans.

Paternal FA deficiency may also influence offspring reproductive development through alterations in sperm quality and epigenetic programming. However, direct evidence linking paternal FA deficiency to reproductive dysfunction in offspring remains limited, and no specific study has established a causal relationship between paternal FA deficiency and offspring reproductive health. Altered sperm DNA methylation has been reported following paternal folate deficiency, raising the possibility that such epigenetic changes may influence embryonic development and subsequent reproductive programming [54]. However, whether these sperm epigenetic alterations directly mediate offspring reproductive phenotypes remains to be determined.

Overall, current evidence suggests that both insufficient and excessive paternal FA exposure may affect sperm quality and epigenetic regulation, but the direction and magnitude of these effects vary according to species, dose, exposure duration, and experimental design. Evidence for direct effects on offspring reproductive health remains limited. Therefore, current findings provide mechanistic insights from preclinical models but are insufficient to establish specific recommendations for paternal FA intake in humans.

### 4.2. Effects of Paternal FA Intake on Embryonic Development

Both insufficient and excessive paternal FA exposure have been associated with adverse embryonic outcomes in animal models. Lifetime paternal exposure to FA deficiency or supplementation was associated with reduced embryo attachment, impaired embryo growth, and increased postnatal pre-weaning mortality in mice [55]. Similarly, another study showed that in mice, paternal FA deficiency increased birth defects in offspring, including craniofacial and musculoskeletal malformations. Genes involved in developmental and chronic diseases (such as cancer, diabetes mellitus, autism, and schizophrenia) in sperm showed differences in methylation compared with controls. FA supplementation in the diet of roosters changed the expression of miRNAs and long non-coding RNAs in sperm and liver of offspring by regulating the expression of key genes involved in glycolysis, gluconeogenesis, and PPAR signaling (*PEPCK*, *ANGPTL4*, *THRSP*) in offspring, which may contribute to disrupted lipid and glucose metabolism and impaired embryonic development. This is detrimental to embryonic development [57]. Alterations in paternal nutritional status have also been associated with changes in sperm and embryonic epigenetic profiles; however, the extent to which these alterations contribute directly to embryonic developmental phenotypes remains unclear. These findings suggest that methyl-donor availability may influence embryonic development and fetal health under paternal nutritional restriction (Figure 4). Another study indicated that some adverse effects associated with paternal FA deficiency were attenuated by maternal FA supplementation during gestation. In rats, impaired glucose tolerance and reduced pancreatic islet β-cell density in female offspring were improved with maternal FA supplementation during gestation, while abnormal hepatic expression of *Ppara*, *Lcn2*, and *Tmcc2* and altered hepatic DNA methylation patterns were restored toward control levels [58]. These results suggest that maternal FA supplementation during gestation may partially mitigate some metabolic consequences associated with paternal FA deficiency [58].

### 4.3. Effects of FA in Paternal Diet on Cognitive Function of Offspring

Emerging evidence indicates that paternal FA and methyl-donor status during the preconception period may influence neurodevelopmental outcomes in offspring. In rats, paternal methyl-donor depletion for five weeks before mating increased anxiety- and depressive-like behaviors in the progeny [59]. Similar findings have been reported in mice, where offspring derived from fathers maintained on a methyl-donor-deficient diet containing folate, methionine, and choline from 3 to 12 weeks of age exhibited enhanced anxiety-related behavior and altered memory-related gene expression [60]. Notably, a paternal methyl-donor-rich diet before conception impaired hippocampus-dependent learning and memory in the offspring and was associated with disrupted hippocampal synaptic plasticity [61]. Current evidence suggests that these neurobehavioral alterations may be associated with epigenetic dysregulation. Reduced paternal FA intake has been associated with altered sperm methylation at loci related to developmental regulation [54]. In parallel, offspring from methyl-donor-deficient fathers exhibit increased promoter methylation of hippocampal memory-related genes such as *Camk2α* and *PP1* [60]. Paternal methyl-donor-rich intake likewise altered epigenetic regulation in the offspring brain, including changes in methylation of the hippocampal Kcnmb2 promoter [61]. One study further reported that restoration of Kcnmb2 expression partially rescued the observed phenotype. Although these findings provide mechanistic insight, independent validation is still lacking [62]. In addition to locus-specific effects, paternal FA deficiency before mating has been shown alter global DNA methylation and Insulin-like Growth Factor 2 (*IGF2*) expression in the fetal brain of offspring [63]. Collectively, these findings suggest that paternal FA and methyl-donor status may influence offspring neurodevelopment and cognitive or behavioral outcomes, although the optimal paternal FA intake range remains to be established (Figure 4).

Overall, current evidence suggests that paternal FA status may influence offspring development through alterations in sperm epigenetic regulation and one-carbon metabolism. However, available evidence is primarily derived from rodent and avian models, with substantial differences in dose, duration, and experimental design among studies. Therefore, these findings should be interpreted as mechanistic insights rather than direct recommendations for human paternal supplementation.

## 5. Paternal Diet and Epigenetics

Although accumulating evidence suggests that paternal diet is associated with alterations in the sperm epigenome, most available evidence is derived from animal models, and only a limited number of studies have provided experimental evidence supporting causal roles for specific sperm-derived epigenetic factors. Therefore, the mechanisms discussed below should be considered as proposed pathways supported by different levels of evidence rather than universally established mechanisms. Epigenetics serves as a critical interface between the genome and the environment, enabling environmental stimuli to induce stable molecular and behavioral alterations that ultimately shape long-term physiological, neurodevelopmental, and behavioral outcomes in offspring [64]. Increasing evidence indicates that sperm-borne epigenetic information, including sncRNAs, DNA methylation, and histone modifications, has been implicated in the intergenerational transmission of paternally acquired traits and disease susceptibility [65,66,67,68].

Among the environmental factors capable of reshaping the male germline, paternal nutrition during the preconception period has emerged as a major determinant of offspring health. The male germline remains particularly susceptible to environmental perturbation during adolescence, adulthood, and the preconception period, when epigenetic programming retains substantial plasticity [32]. Nutritional imbalance during this sensitive developmental window can profoundly remodel the sperm epigenome during gametogenesis [32]. Environmental stressors may leave persistent epigenetic signatures within sperm chromatin that have been proposed to influence offspring development [69]. In particular, Paternal HFD exposure induces sperm mitochondrial dysfunction and alters sperm-derived mitochondrial RNAs, which have been implicated in metabolic programming of offspring [70,71]. Mechanistically, HFD promotes the accumulation of fragmented mitochondrial tRNAs (mt-tRNAs) in mature sperm. These sperm-derived mt-tRNAs are delivered to the oocyte at fertilization and may influence early embryonic metabolic gene regulation involved in oxidative metabolism during early embryogenesis. Such alterations may contribute to glucose intolerance observed in offspring in life [71]. These findings support the concept that sperm RNAs are highly responsive to metabolic stress and may function as rapid carriers of environmentally induced paternal information [72].

Compared with the dynamic responsiveness of sperm RNAs, DNA methylation and histone-associated modifications may provide greater epigenetic stability across developmental stages. Data from the Neonatal Epigenetics Study (NEST) cohort demonstrated that paternal obesity was associated with altered methylation of the imprinted *IFG2* locus in newborns [73]. Similarly, paternal HFD in mice was associated with concordant methylation changes within the *IFG2*-*H19* imprinting control regions in both paternal sperm and offspring liver tissues, suggesting a possible germline-mediated contribution [74]. Recent studies have extended these observations to epitranscriptomic regulation. One study reported that multigenerational paternal obesity was associated with susceptibility to spermatogenic dysfunction through Methyltransferase-like 3 (METTL3) mediated N6-methyladenosine modification of Wt1 [75]. In parallel, paternal HFD induced similar alterations in DNA methylation and miRNA profiles in the sperm epigenome of both fathers and their male offspring. Notably, paternal HFD exposure has been reported to alter sperm miRNA profiles, which may contribute to the transmission of metabolic phenotypes to offspring [76]. However, whether these findings represent true transgenerational inheritance requires further validation. Together, these findings suggest that paternal dietary exposure can reprogram the sperm epigenome and may influence offspring metabolic and reproductive phenotypes. Evidence for persistence across multiple generations remains limited [77]. However, whether these DNA methylation changes are sufficient to mediate offspring phenotypes independently remains to be fully established.

Following fertilization, both sperm and oocytes undergo extensive epigenetic reprogramming to establish a totipotent zygotic state capable of generating all embryonic lineages. This process involves large-scale chromatin remodeling, histone modification, and zygotic genome activation. Consequently, a major unresolved question in the field is how environmentally induced paternal epigenetic information escapes this widespread reprogramming process. Among the candidate mechanisms, Histone H3 lysine 4 trimethylation (H3K4me3) has received considerable attention. Loss of sperm H3K4me3 has been associated with abnormal embryonic gene expression and congenital defects in offspring. Nutritional perturbations, including paternal folate deficiency, have been reported to influence sperm H3K4me3 landscapes, with some of these alterations detectable in early embryos and associated with developmental abnormalities [69]. These findings raise the possibility that altered sperm H3K4me3 may contribute to developmental programming, although a direct causal relationship remains to be established. Accumulating evidence further suggests that H3K4me3 exerts distinct biological functions depending on its enrichment level. Highly enriched H3K4me3 promoters are primarily associated with spermatogenesis and sperm maturation, including pathways related to transcriptional regulation, protein modification, RNA splicing, and mitophagy. Moderately enriched regions are linked to cell division and embryonic development, whereas loci with lower enrichment are more closely associated with intracellular trafficking and signaling pathways [78].

Although the transgenerational role of H3K4me3 has been demonstrated in yeast, flies, and worms, the mechanisms by which specific H3K4me3 signatures evade epigenetic reprogramming during embryonic and germline development remain poorly understood. Notably, obesity-associated H3K4me3 alterations are enriched at loci involved in placental development and inflammatory signaling pathways. Dysregulated inflammatory gene expression within the placenta has been linked to hypertensive pregnancy disorders [79]. These observations raise the possibility that H3K4me3 may represent one mechanism linking paternal nutritional status with offspring phenotypes through placental regulation; however, direct causal evidence remains limited [79]. Collectively, these findings highlight the emerging role of non-DNA-based inheritance mechanisms in mediating the long-term biological consequences of paternal nutritional exposure. Similarly, evidence supporting the functional transmission of altered histone modifications remains limited and is derived predominantly from rodent studies.

Current evidence supports epigenetic remodeling as a central mechanism underlying the association between paternal diet and offspring development. The relative stability of epigenetic marks may explain how environmentally induced molecular alterations persist throughout life and influence metabolic pathways, endocrine homeostasis, and developmental programming in subsequent generations [79]. Despite substantial progress, several key questions remain unresolved, particularly regarding which epigenetic signatures retain sufficient stability to escape embryonic reprogramming and mediate long-term inheritance.

Increasing evidence suggests that DNA methylation, histone modifications, sncRNAs, and RNA modifications function as interconnected rather than independent epigenetic regulators within the male germline [68]. These mechanisms exhibit extensive cross-talk, whereby chromatin organization influences DNA methylation landscapes and sperm RNA composition, while sperm-borne RNAs and RNA modifications may further modulate chromatin remodeling and early embryonic gene expression after fertilization. Rather than acting as isolated pathways, these epigenetic processes are therefore thought to form an integrated regulatory network that collectively influences zygotic genome activation and developmental programming. However, the molecular interactions among these pathways, as well as their relative contributions to offspring phenotypes, remain incompletely understood and require further functional validation. Several candidate sperm-borne small RNAs have been identified, although many have been reported in single studies and require independent validation. Addressing these questions will be essential for understanding how paternal nutrition shapes disease susceptibility across generations and may provide new opportunities for preventing metabolic, reproductive, and developmental disorders [68].

Despite considerable progress, it remains unclear which sperm-derived epigenetic marks can consistently escape post-fertilization epigenetic reprogramming and whether these marks directly contribute to offspring phenotypes. Furthermore, most published studies have examined only the F1 generation; therefore, the evidence summarized in this review primarily supports intergenerational rather than transgenerational inheritance. Additional functional studies are required to validate the causal roles of specific epigenetic mechanisms and their relevance to human health. It is important to distinguish intergenerational from transgenerational inheritance. In this review, intergenerational effects refer to phenotypic alterations observed in directly exposed offspring (F1), whereas transgenerational inheritance requires persistence of phenotypes in subsequent generations without continued exposure. Since most paternal diet studies evaluate F1 offspring, the current evidence predominantly supports intergenerational effects rather than reported transgenerational inheritance.

## 6. Conclusions and Perspectives

### 6.1. Current Evidence and Limitations of Paternal Nutritional Programming

Accumulating evidence from preclinical studies indicates that paternal nutritional status before conception is associated with offspring reproductive, developmental, metabolic, and neurobehavioral outcomes. Experimental studies suggest that paternal dietary patterns, including HFD, LPD, and altered folate status, may influence offspring phenotypes through changes in sperm quality and epigenetic regulation. However, corresponding human evidence remains limited and is largely observational, highlighting the need for cautious interpretation of current findings.

Despite substantial progress, several important questions remain unresolved. Current evidence is derived predominantly from rodent models, with substantial heterogeneity in species, dietary composition, nutrient dose, exposure duration, and experimental design. In addition, reproductive physiology, spermatogenic characteristics, and nutritional requirements differ considerably among experimental species, including mice, rats, and poultry. Therefore, findings from different animal models should be interpreted within their specific biological context rather than as evidence of a single conserved mechanism. Furthermore, many proposed mechanisms remain supported by associative evidence or individual studies, and it remains unclear which sperm epigenetic alterations can escape post-fertilization reprogramming and directly contribute to offspring phenotypes. Furthermore, because most studies have evaluated only directly exposed F1 offspring and relatively few have extended analyses to subsequent generations, current evidence primarily supports intergenerational rather than reported transgenerational inheritance.

Although dietary improvement, physical activity, and other nutritional interventions have shown potential effects in preclinical studies, their efficacy and safety for improving paternal reproductive outcomes remain to be validated in humans. Therefore, current findings should not be directly translated into dietary recommendations for prospective fathers.

### 6.2. Convergent and Divergent Molecular Pathways Across Dietary Patterns

A cross-comparison of the three dietary paradigms reveals both common and distinct intergenerational signaling axes. Consistently, HFD, LPD, and FA imbalance have been reported to influence one-carbon metabolism and DNA methylation-related pathways, although the specific effects vary among dietary models. However, the direction and locus-specificity of methylation changes differ: HFD has been associated with altered methylation patterns at neurotrophic genes, including *BDNF*, in specific animal models, whereas LPD is associated with global hypomethylation and reduced expression of methyltransferases in the testis. In terms of metabolic pathways, HFD has been primarily associated with alterations in mitochondrial function and lipid metabolism, while LPD has been reported to affect amino acid transport and nutrient-sensing pathways, including mTOR-related signaling. FA imbalance has been reported to influence histone methylation dynamics, including H3K4me3 regulation, RNA modification pathways, and RNA methylation, suggesting a broader impact on transcriptional and post-transcriptional regulation. These distinctions imply that while all three nutritional stressors operate through the germline, each dietary challenge may produce distinct epigenetic signatures that translate into partially overlapping offspring phenotypes. This duality supports the concept that paternal nutrition acts not through a single universal mechanism, but rather through a network of interacting pathways that are differentially engaged depending on the nature of the nutritional challenge.

### 6.3. Future Directions and Translational Challenges

Despite substantial progress, the mechanistic basis of paternal dietary programming remains incompletely understood. Most current studies have focused on individual epigenetic mechanisms, including DNA methylation, histone modifications, and non-coding RNAs. However, how these pathways interact within sperm and whether specific epigenetic signatures can escape embryonic reprogramming after fertilization remain unresolved. Future studies integrating epigenomics, transcriptomics, metabolomics, and chromatin profiling, and functional validation approaches will be essential to clarify the causal relationships between paternal nutrition, sperm epigenetic alterations, and offspring phenotypes.

Another major challenge is the limited translational evidence in humans. Although experimental studies in rodents have provided important mechanistic insight, their relevance to human populations remains uncertain. Large-scale prospective birth cohorts incorporating standardized paternal dietary assessments longitudinal sperm epigenome profiling, and offspring health follow-up are required to determine whether specific paternal dietary patterns are consistently associated with offspring outcomes. Such studies may also facilitate the identification of sperm epigenetic biomarkers predictive of developmental or metabolic risk.

## Data Availability

No new data were created or analyzed in this study. Data sharing is not applicable to this article.

## Supplementary Materials

The following supporting information can be downloaded at: https://www.mdpi.com/article/10.3390/nu18183008/s1, Table S1: Experimental exposure parameters and offspring outcomes in studies of paternal dietary imbalance; Table S2: Potential intervention strategies for mitigating adverse effects associated with paternal dietary imbalance.

## Abbreviations

The following abbreviations are used in this manuscript:

HFD	High-fat diet
LPD	Low-protein diet
FA	Folic acid
BDNF	Brain-derived neurotrophic factor
NEST	Neonatal Epigenetics Study
sncRNAs	small non-coding RNAs
TNF-α	tumor necrosis factor
mt-tRNAs	mitochondrial tRNAs
DNMT1/DNMT3L	DNA methyltransferases 1, 3-like
IGF2	Insulin-like Growth Factor 2
METTL3	Methyltransferase-like 3
H3K4me3	Histone H3 lysine 4 trimethylation
