# Supplementary material for: Intergenerational Effects of Paternal Diets on Offspring Health and Reproduction
Source: Nutrients. 2026 Sep 14;18(18):3008. doi: 10.3390/nu18183008 (PMC13610363; doi:10.3390/nu18183008)
Supplement: Supplementary file 1 [file nutrients-18-03008-s001.zip › nutrients-4462625-supplementary.pdf]

Table S1. Experimental exposure parameters and offspring outcomes in studies of paternal dietary imbalance

Dietary model	Model	Diet composition / dose	Exposure duration	Outcomes	Reference
HFD	Male mice	30% fat	19 wk	No apparent impairment of fertility	[19]
HFD	Male mice	60% kcal from fat	12 wk	Sperm quality; altered offspring phenotypes↓	[24]
LPD	Male mice	9% protein ± methyl donors	≥7 wk	Altered testicular physiology	[51]
LPD	Male mice	9% casein	≥8 wk	Offspring adiposity and glucose intolerance↑	[54]
FA deficiency	Male mice	0.3 mg FA/kg diet	2–10 wk	Sperm count↓; adverse pregnancy outcomes	[65]
FA excess	Male mice	20–40 mg FA/kg diet	10 wk	Sperm count at high exposure↓	[65]

Table S2. Potential intervention strategies for mitigating adverse effects associated with paternal dietary imbalance.

Intervention strategy	Targeted paternal diet	Model system	Outcomes	References
Probiotic supplementation	HFD	Obese male mice	Improved testosterone and sperm quality	[26,27]
Paternal exercise	HFD	Male mice and offspring	Improved offspring metabolic phenotypes	[30]
Paternal exercise	HFD	Male mice and offspring	Altered placental inflammation and nutrient transporter expression; modified fetal weight	[31]
Probiotic supplementation	HFD	Male mice	Improved testicular function and sperm quality	[32]
Dietary improvement / metabolic intervention	Metabolic dysfunction	Human/animal models	Improved insulin sensitivity, blood pressure and cardiometabolic parameters	[43,44]
Methyl-donor supplementation	LPD	Male mice	Partially restored testicular physiology	[51,52]
